# Comparative transcriptome analysis of root, stem, and leaf tissues of *Entada phaseoloides* reveals potential genes involved in triterpenoid saponin biosynthesis

**DOI:** 10.1186/s12864-020-07056-1

**Published:** 2020-09-15

**Authors:** Weifang Liao, Zhinan Mei, Lihong Miao, Pulin Liu, Ruijie Gao

**Affiliations:** 1 School of biology and pharmaceutical engineering, Wuhan Polytechnic University, Wuhan, 430023 People’s Republic of China; 2 School of Pharmacy, South-Central University for Nationalities, Wuhan, 430074 People’s Republic of China

**Keywords:** *Entada phaseoloides*, Transcriptome, Triterpenoid saponins, Secondary metabolites

## Abstract

**Background:**

*Entada phaseoloides* (L.) Merr. is an important traditional medicinal plant. The stem of *Entada phaseoloides* is popularly used as traditional medicine because of its significance in dispelling wind and dampness and remarkable anti-inflammatory activities. Triterpenoid saponins are the major bioactive compounds of *Entada phaseoloides.* However, genomic or transcriptomic technologies have not been used to study the triterpenoid saponin biosynthetic pathway in this plant.

**Results:**

We performed comparative transcriptome analysis of the root, stem, and leaf tissues of *Entada phaseoloides* with three independent biological replicates and obtained a total of 53.26 Gb clean data and 116,910 unigenes, with an average N50 length of 1218 bp. Putative functions could be annotated to 42,191 unigenes (36.1%) based on BLASTx searches against the Non-redundant, Uniprot, KEGG, Pfam, GO, KEGG and COG databases. Most of the unigenes related to triterpenoid saponin backbone biosynthesis were specifically upregulated in the stem. A total of 26 cytochrome P450 and 17 uridine diphosphate glycosyltransferase candidate genes related to triterpenoid saponin biosynthesis were identified. The differential expressions of selected genes were further verified by qPT-PCR.

**Conclusions:**

The dataset reported here will facilitate the research about the functional genomics of triterpenoid saponin biosynthesis and genetic engineering of *Entada phaseoloides*.

## Background

*Entada phaseoloides* (L.) Merr. is a liana belonging to Fabaceae family. It grows in Southern China and other tropical countries. The stem of *Entada phaseoloides* is popularly used in traditional medicine because of its significant pharmacological activities [1–3]. The stem of *Entada phaseoloides*, also called “Guo Gang Long,” produces curative effects that dispel wind and dampness and exhibits remarkable anti-inflammatory activity. Its main bioactive ingredients are triterpenoid saponins compounds [3]. Various types of triterpene saponins have been isolated from *E. phaseoloides*. The representative saponins of *Entada phaseoloides* are oleanane-type triterpene saponins which contain seven sugar chains.

The mevalonic acid (MVA) pathway is an important metabolic pathway in plants [4, 5]. Triterpenoid saponins comprise six isoprene units and are derived from a C-30 hydrocarbon precursor, squalene. Squalene is synthesized from isopentenyl diphosphate (IPP) via the MVA pathway. Subsequently, squalene epoxidase (SQE) catalyzes the conversion of squalene to 2,3-oxidosqualene. The diversifying step in triterpenoid backbone biosynthesis is the cyclization of 2,3-oxidosqualene catalyzed by a class of oxidosqualene cyclases (OSCs) [6, 7]. Cytochrome P450 monooxygenases (CYP450s) and UDP-glycosyltransferases (UGTs) govern the hydroxylation, oxidation, and glycosylation steps, yielding triterpenoid saponins [8–10]. However, the key genes related to triterpenoid saponin biosynthesis in *Entada phaseoloides* have not been identified.

High-throughput sequencing analysis is a useful method to clarify the molecular mechanism of plant secondary metabolism [11, 12]. Recently, transcriptome assay with next-generation sequencing has been extensively used to explore the novel genes underlying active-ingredient biosynthesis pathways in medicinal plants. Some include the excavation of genes encoding enzymes that catalyze distinct steps related to the biosynthetic pathway of ginsenosides in *Panax ginseng* [13], triterpenoid saponin biosynthesis in *Bacopa monnieri* [14], artemisinin in *Artemisia annua* [15, 16], flavonoid biosynthesis in safflower [17], glycyrrhizin in *Glycyrrhiza uralensis* [18], rubber in *Parthenium argentatum* [19]*,* cardiac glycoside in *Calotropis procera* [20], terpenoid in *Cinnamomum camphora* [21], cannabinoids in *Cannabis sativa* [22], withanolide in *Withania somnifera* [23], picrosides in *Picrorhiza kurrooa* [24], paclitaxel in *Taxus chinensis* [25] and steroidal saponins in *Asparagus racemosus* [26].

Because the synthesis and accumulation of specific metabolites in different tissues depends on the age of the plant, and are greatly affected by the different developmental stages. It was found that the content of triterpenes accumulated in the leaves of *P. ginseng* are higher in the early growth stage, while the content of triterpenes in the roots of old plants are higher [27]. Comparative transcriptome analysis including root and leaf tissues to excavate transcripts related to saponin biosynthesis is already reported for many plants, such as *Hedera helix* [28], *Panax notoginseng* [29] and *Asparagus racemosus* [26]. The maximum triterpenoid saponin content was identified in stem. So, in this study, comparative transcriptome analysis of root, stem, and leaf tissues of *Entada phaseoloides* was performed to identify genes related to triterpenoid saponin. We obtained thousands of putative genes, including a series of genes related to triterpene saponin biosynthesis. Moreover, different expression patterns of CYP450s and UGTs in the three tissues were analyzed. This work was established to functionally research the genes related to triterpene saponin biosynthesis and provide more information about this species.

## Results

### Illumina sequencing and de novo assembly

To characterize the transcriptomes of *Entada phaseoloides*, we sequenced nine cDNA libraries prepared from the root, stem, and leaf tissues with three biological repeats by using the Illumina Hiseq 2500 platform. A total of 53.26 Gb clean data were obtained after removing adaptors, poly-A tails, and primer sequences, short (< 50 bp), and low-quality sequences. A total of 57–60 million for each tissue were generated (Additional file 1). The high-quality reads were assembled using the Trinity program [30] and the TGI clustering tool (TGICL) [31] to remove redundant sequences. Finally, 116,910 unigenes were identified, with an average N50 length of 1218 bp (Additional file 1). The correlation indices between repeated samples were > 0.9 (Additional file 2), indicating that the Illumina sequencing results are credible.

### Functional annotation

**Fig. 1:**
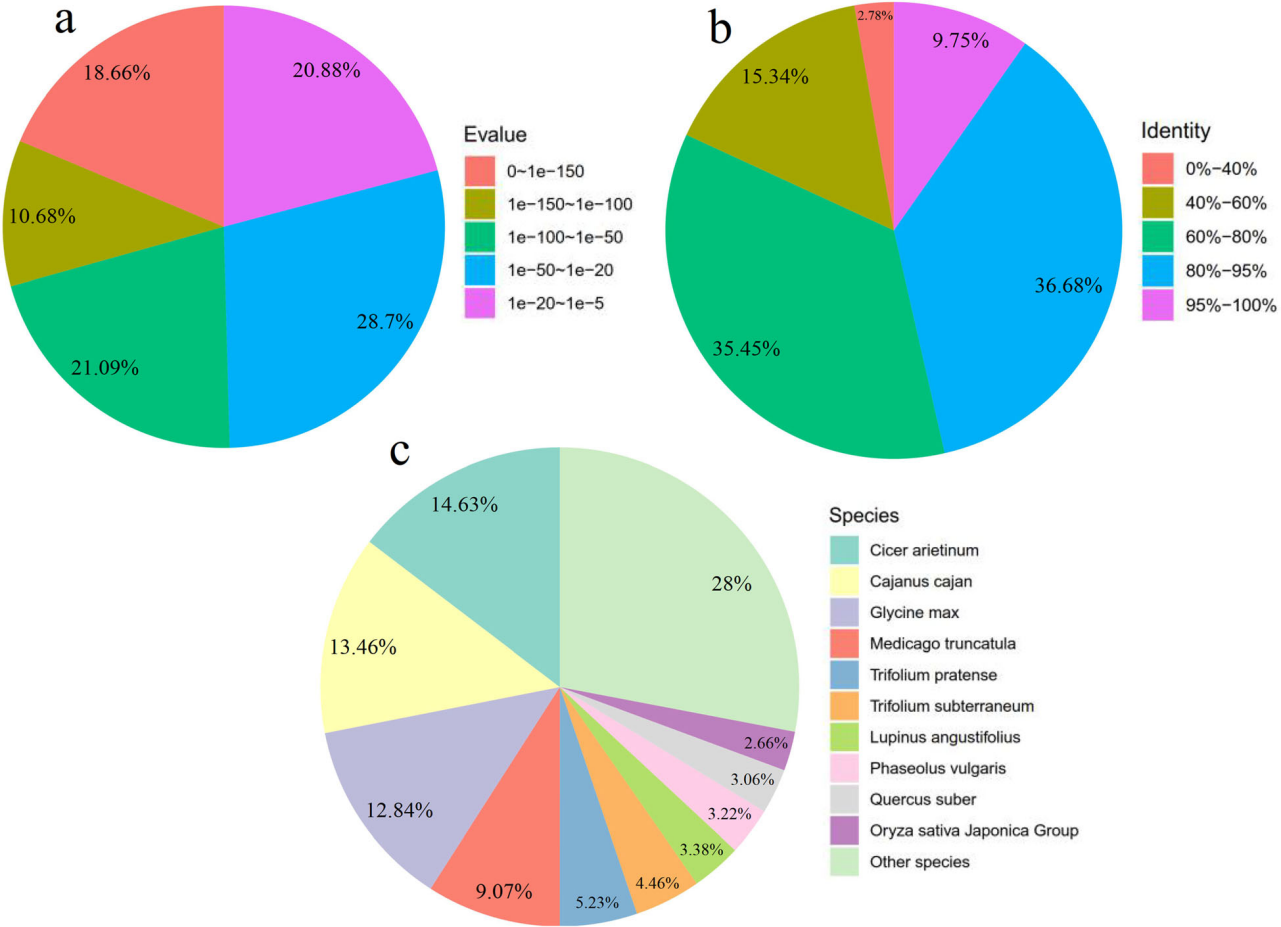
Similarity of unigenes annotated using the Nr database. **a** E-value distribution of best BLAST hits for each unigene (E-value <1e-5). **b** Similarity distribution of top BLAST hits for each unigene. **c** Distribution of the most homologous sequence results for each unigene by species (E-value <1e-5)

All assembled unigenes were searched against the Non-redundant (Nr), Uniprot, Kyoto Encyclopedia of Genes and Genomes (KEGG), Pfam, Gene Ontology (GO), and Clusters of Orthologous Groups (COG) databases using the BLASTx program with *E*-value <1e-5. Among the 116,910 sequences, 42,191 (36.1%), 41,228 (35.3%), 28,126 (24.1%), 26,874 (23.0%), 15,119 (13.0%) and 11,812 (10.1%) unigenes showed significant similarity to known proteins in NR, Uniprot, GO, Pfam, KEGG and COG database, respectively. The result of BLASTX with different databases and their annotation were listed in Additional file 3. Based on the Nr database, the E-value distribution indicated that 70.67% of the matched unigenes ranged from 1e-5 to 1e-100 (Fig. 1a). For the similarity distribution, 46.43% unigenes exhibited a similarity of above 80%, whereas 50.79% of the unigenes showed a similarity of 40–80% (Fig. 1b). Furthermore, 14.63% of the *Entada phaseoloides* unigenes shared high similarity with the genes of *Cicer arietinum*, 13.46% similarity with *Cajanus cajan*, 12.84% similarity with *Glycine max*, and 9.07% similarity with *Medicago truncatula* (Fig. 1c).

GO analysis included three main domains that describe biological processes, cellular components, and molecular functions. When GO was used to classify gene functions, 28,126 unigenes were assigned to 60 functional categories (Additional files 4 and 5). Within the biological process domain, the three most enriched categories were “biosynthetic process,” “cellular nitrogen compound metabolic process,” and “response to stress.” In the cellular component domain, the three most matched categories were “cellular component.” “nucleus,” and “protein complex.” In the molecular function domain, the three most common categories were “ion binding,” “molecular function,” and “kinase activity.”

**Fig. 2:**
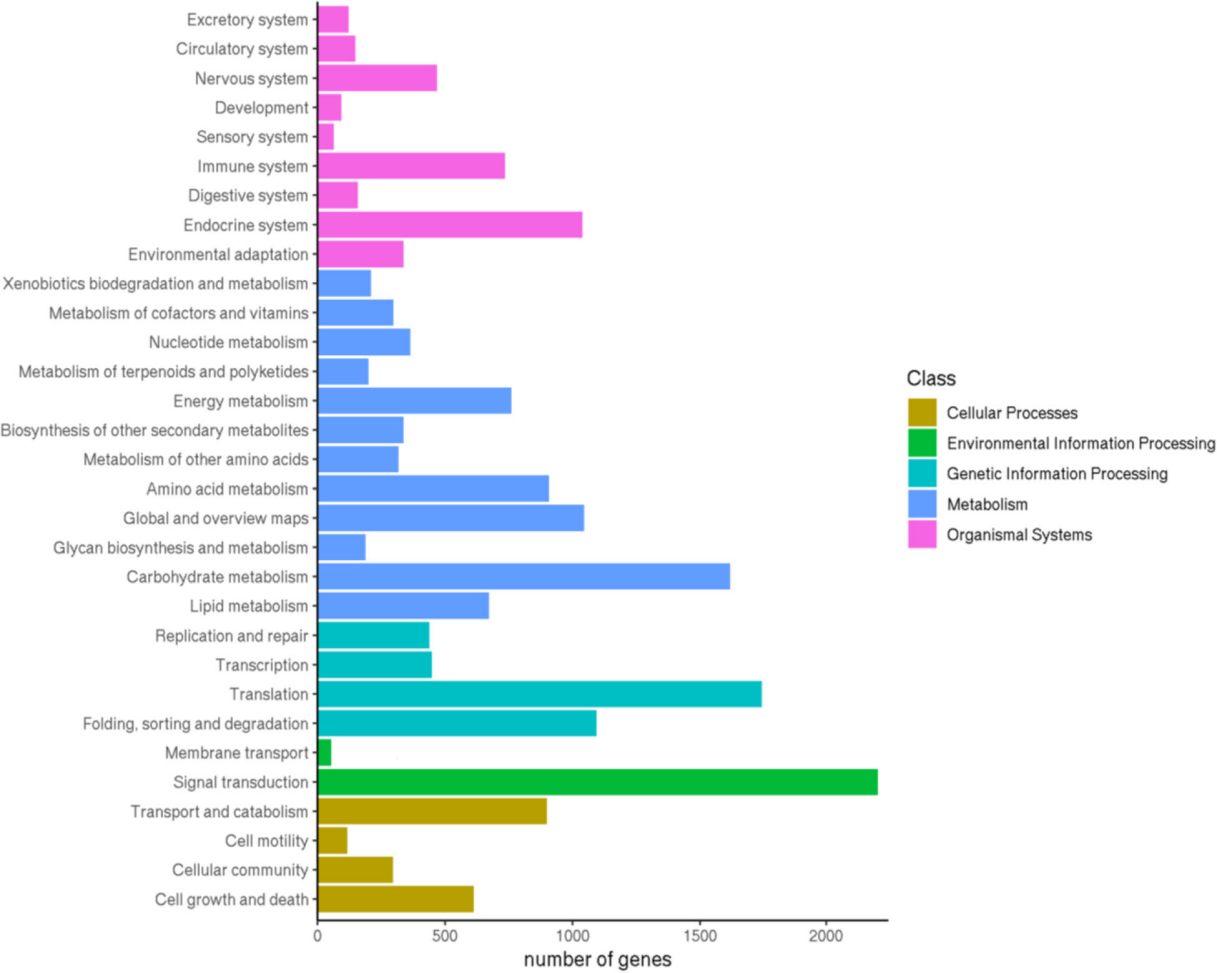
KEGG pathway classification of *Entada phaseoloides* unigenes. Exactly 15,119 unigenes were divided into five main categories in accordance with the corresponding pathways

To better understand the functions of specific metabolic pathways in *Entada phaseoloides*, we mapped the annotated unigenes to the reference biological pathways in the KEGG database. A total of 15,119 unigenes (13.0%) could be assigned to five main categories and 31 sub-categories (Fig. 2, Additional file 6). These enzymes feature assigned functions in 28 secondary metabolic pathways in KEGG (Table 1). Among these unigenes, 147 encode key enzymes are related to the pathways for terpenoid biosynthesis, including the synthesis of the terpenoid backbone (63 unigenes), monoterpenoids (7 unigenes), diterpenoids (18 unigenes), sesquiterpenoids and triterpenoids (16 unigenes), and other terpenoid-quinone complexes (43 unigenes). Fifty unigenes are involved in alkaloid biosynthesis, including isoquinoline alkaloid (24 unigenes) and tropane, piperidine, and pyridine alkaloid biosynthesis (26 unigenes). Exactly 210 unigenes were associated with the flavonoid biosynthesis pathway, including the phenylpropanoid (162 unigenes), flavonoid (36 unigenes), flavone and flavonol (7 unigenes), and isoflavonoid (5 unigenes) biosynthesis pathways. Unigenes involved in these pathways should be further identified to understand their functions in the biosynthesis of active ingredients in leguminous plants.

**Table 1:** Secondary metabolism pathways in *Entada phaseoloides*

Pathway ID	Pathways	Unigene number
ko00100	Steroid biosynthesis	33
ko00130	Ubiquinone and other terpenoid-quinone biosynthesis	43
ko00232	Caffeine metabolism	6
ko00254	Aflatoxin biosynthesis	1
ko00261	Monobactam biosynthesis	19
ko00332	Carbapenem biosynthesis	1
ko00400	Phenylalanine, tyrosine and tryptophan biosynthesis	58
ko00401	Novobiocin biosynthesis	2
ko00521	Streptomycin biosynthesis	23
ko00524	Butirosin and neomycin biosynthesis	12
ko00860	Porphyrin and chlorophyll metabolism	49
ko00900	Terpenoid backbone biosynthesis	63
ko00902	Monoterpenoid biosynthesis	7
ko00903	Limonene and pinene degradation	21
ko00904	Diterpenoid biosynthesis	18
ko00905	Brassinosteroid biosynthesis	13
ko00906	Carotenoid biosynthesis	26
ko00908	Zeatin biosynthesis	18
ko00909	Sesquiterpenoid and triterpenoid biosynthesis	16
ko00940	Phenylpropanoid biosynthesis	162
ko00941	Flavonoid biosynthesis	36
ko00943	Isoflavonoid biosynthesis	5
ko00944	Flavone and flavonol biosynthesis	7
ko00945	Stilbenoid, diarylheptanoid and gingerol biosynthesis	11
ko00950	Isoquinoline alkaloid biosynthesis	24
ko00960	Tropane, piperidine and pyridine alkaloid biosynthesis	26
ko00965	Betalain biosynthesis	1
ko00966	Glucosinolate biosynthesis	1

### Differentially expressed gene (DEG) analysis

**Fig. 3:**
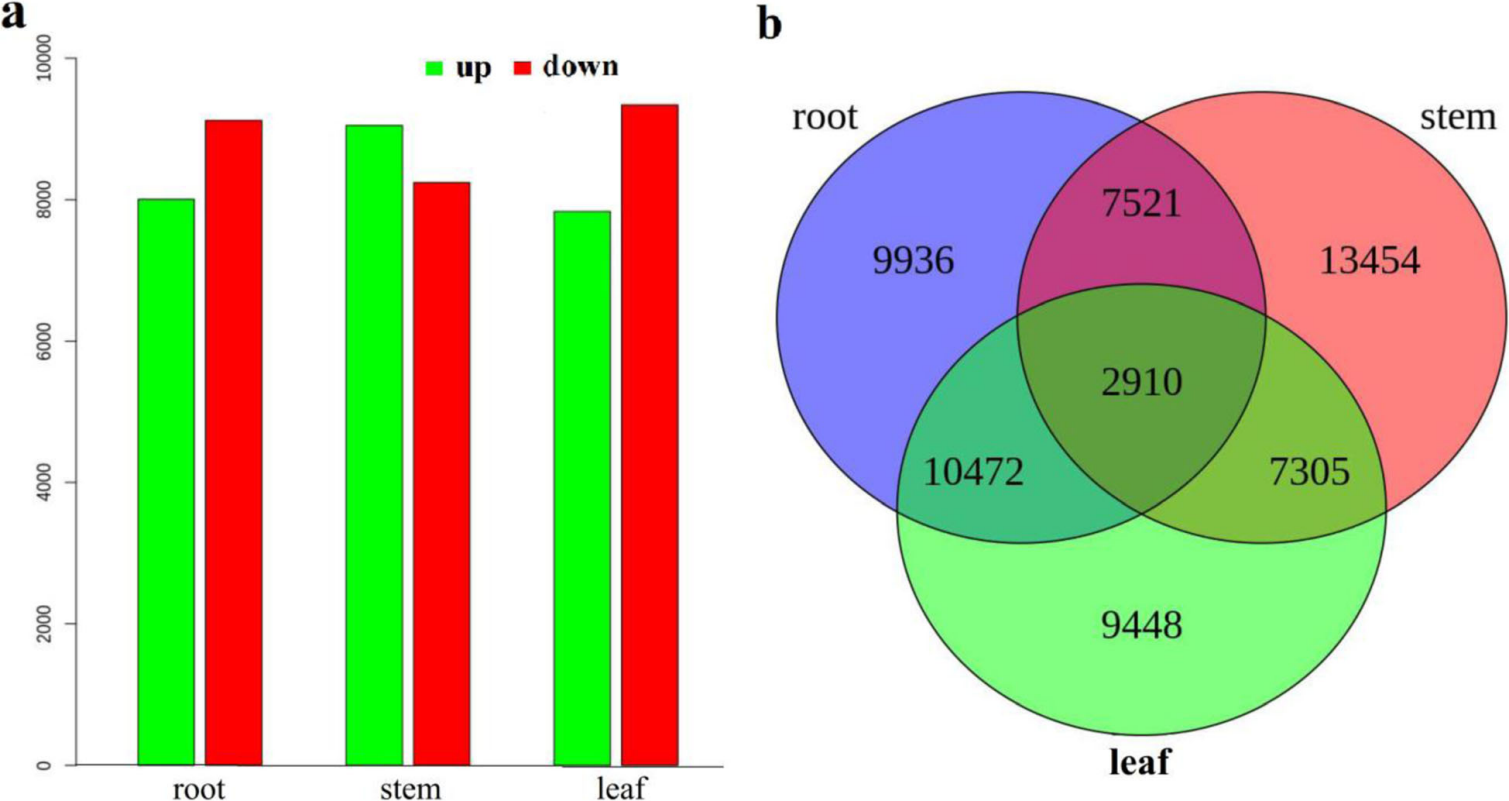
Differential expression analysis of unigenes. **a** Number of DEGs in each tissue compared with the other two tissues; **b** Venn diagram representing the number of DEGs among *Entada phaseoloides* tissues

The clean reads were mapped back onto the assembled unigenes by using the alignment via Burrows–Wheeler aligner (BWA) program to analyze the DEGs among different tissues [32]. The Fragments per Kilobase Million (FPKM) value was calculated for each unigene in each tissue of *Entada phaseoloides*. The DEGs were identified (Additional file 7) using FDR ≤ 0.001 and |log2Ratio| ≥ 1 [33]. The lowest number of DEGs was observed between the stem and leaf tissues, and the highest was noted between the root and leaf. Furthermore, the DEGs in one tissue were studied and compared with those in the other two tissues. The stem contained the largest number of highly expressed unigenes, having 8962 unigenes more abundant in the stem. Figure 3 shows the other expression differences among various tissues.

**Fig. 4:**
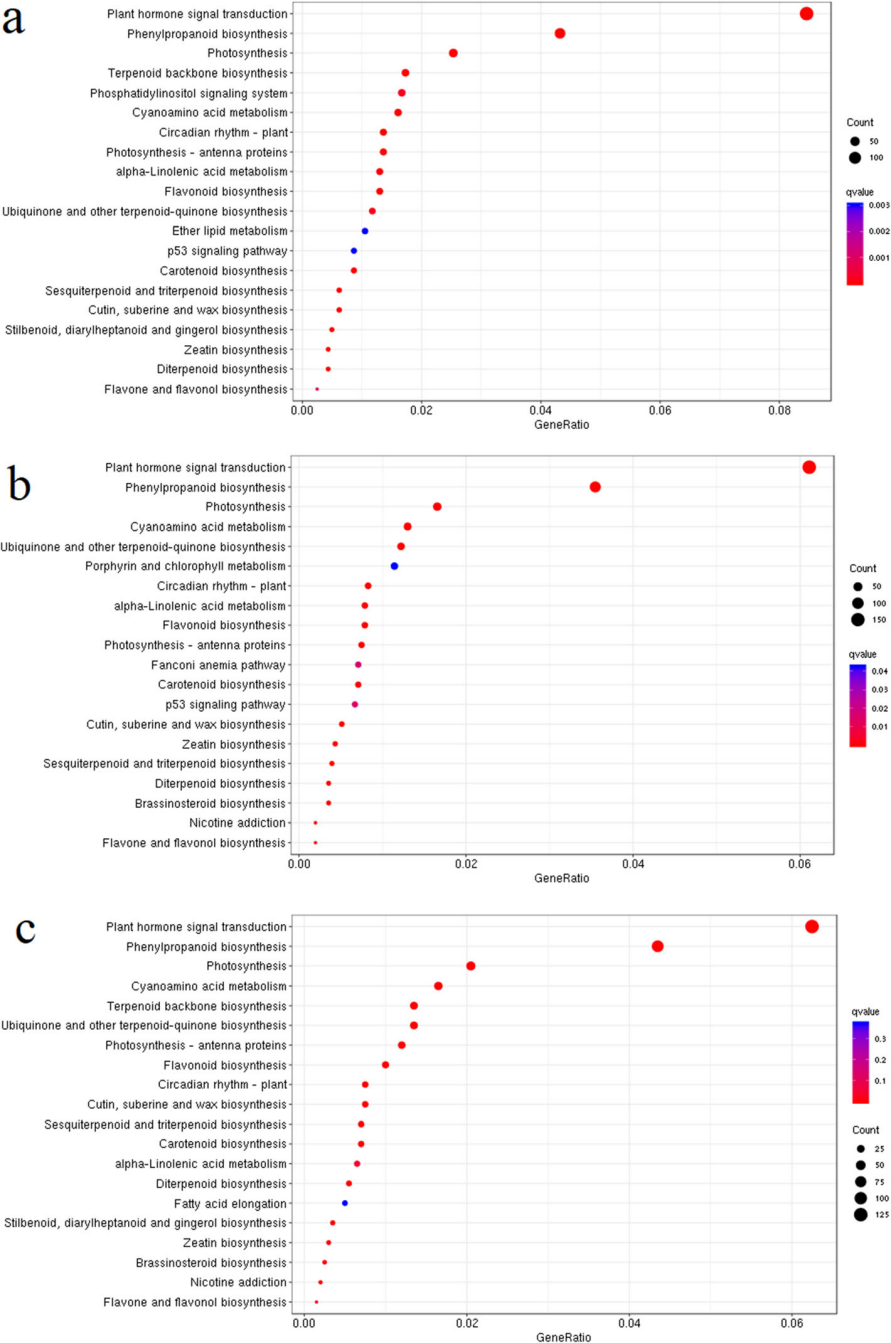
KEGG enrichment analyses of DEGs in different tissues. **a** between leaf and stem; **b** between leaf and root; **c** between stem and root

KEGG enrichment analyses were performed with the DEGs among the root, stem, and leaf tissues to investigate the genes regulating the distribution of triterpenoid saponin. These DEGs were evidently enriched in specific pathways. Meanwhile, the top 20 significant pathways were analyzed based on the FDR ≤ 0.01. Between the leaf and stem, plant hormone signal transduction, phenylpropanoid biosynthesis, photosynthesis, terpenoid backbone biosynthesis, and phosphatidyllnositol signaling system showed significant enrichment (Fig. 4a). Between the leaf and root, plant hormone signal transduction, phenylpropanoid biosynthesis, photosynthesis, ubiquinone and other terpenoid-quinone biosynthesis, and cyanoamino acid metabolism pathways showed visible differential expression (Fig. 4b). Between the stem and root, plant hormone signal transduction, phenylpropanoid biosynthesis, photosynthesis, cyanoamino acid metabolism, and terpenoid backbone biosynthesis showed significant enrichment (Fig. 4c). In addition to the common pathways of primary metabolism, enriched secondary metabolic pathways, including terpenoid and phenylpropanoid biosynthesis, were also found between different tissues, indicating the possible distinct distribution of secondary metabolites in different tissues.

### Putative genes involved in triterpenoid saponin backbone biosynthesis

**Fig. 5:**
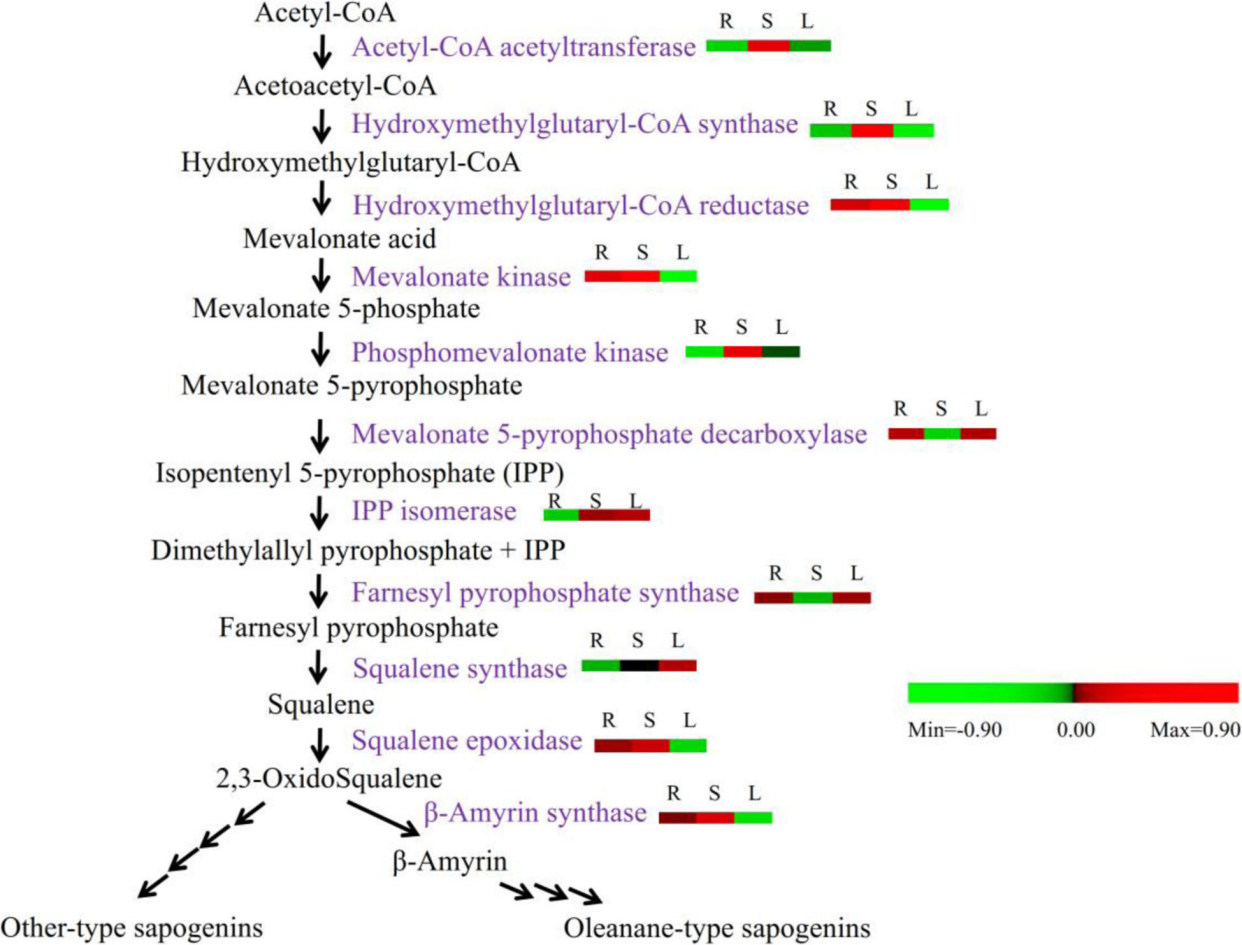
Schematic representation of the potential triterpenoid saponin biosynthesis pathway. Transcriptomic data (lg FPKM) for each gene represent the expression in the root (R), stem (S), and leaf (L) on heat map

Triterpenes are synthetized from a five-carbon isoprene unit through the cytosolic MVA pathway. Triterpenoid saponins are composed of six isoprene units and are derived from the C-30 hydrocarbon precursor, squalene. Squalene is synthesized from isopentenyl diphosphate (IPP) via the MVA pathway. All genes encoding the enzymes associated with the upstream regions of triterpenoid biosynthesis were successfully detected in the *Entada phaseoloides* transcriptome. Their expression value was monitored in three biological replicates along with their mean values (Table 2, Fig. 5). Most unigenes related to MVA pathway were specifically upregulated in the stem tissue. Hydroxymethylglutaryl-CoA reductase showed the highest expression, which is the rate limiting step MVA pathway for saponin biosynthesis.

**Table 2:** List of transcripts related to triterpenoid saponin backbone biosynthesis

Annotation	Enzyme code	Number of Unigenes	Expression value (Root)	Expression value (Stem)	Expression value (Leef)
Acetyl-CoA acetyltransferase	EC: 2.3.1.9	3	75.867	198.683	101.921
Hydroxymethylglutaryl-CoA synthase	EC: 2.3.3.10	4	91.369	386.235	56.173
Hydroxymethylglutaryl-CoA reductase	EC:1.1.1.34	10	450.327	760.578	72.126
Mevalonate kinase	EC:2.7.1.36	6	5.834	15.325	0.428
Phosphomevalonate kinase	EC: 2.7.4.2	1	11.324	40.568	20.753
Mevalonate-5-pyrophosphate decarboxylase	EC: 4.1.1.33	3	96.023	51.040	93.265
Isopentenyl pyrophosphate isomerase	EC: 5.3.3.2	1	20.335	32.018	35.736
Farnesyl pyrophosphate synthase	EC: 2.5.1.10	1	95.416	70.236	99.372
Squalene synthase	EC: 2.5.1.21	5	68.731	84.359	73.837
Squalene epoxidase	EC:1.14.99.7	16	163.378	206.325	90.231
β-Amyrin synthase	EC: 5.4.99.39	8	60.329	91.661	32.965
Lupeol Synthase	EC: 5.4.99.41	1	5.368	2.197	3.562
Cycloartenol synthase	EC: 5.4.99.8	5	10.312	15.385	2.214

**Fig. 6:**
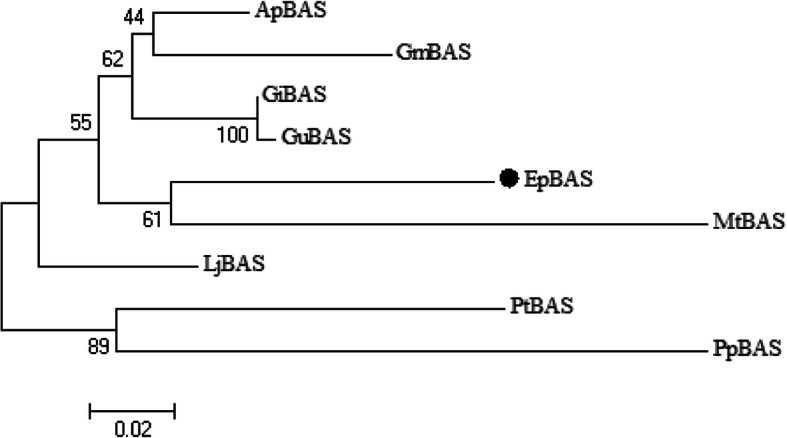
Phylogenetic analysis of the EpBAS and other plant BASs. The distances between each clone and group were calculated using CLUSTAL W. Bootstrap values are shown. Ap: *Abrus precatorius*; Gm: *Glycine max*; Gi: *Glycyrrhiza inflata*; Gu: *Glycyrrhiza uralensis*; Ep: *Entada phaseoloides*; Mt: *Medicago truncatula*; Lj: *Lotus japonicus*; Pt: *Polygala tenuifolia*; Pp: *Prunus persica*

The diversifying step in triterpenoid backbone biosynthesis is the cyclization of 2,3-oxidosqualene catalyzed by a class of OSCs. The major saponins in *Entada phaseoloides* are oleanane-type triterpenoid saponins derived from β-amyrin. The Illumina sequencing of *Entada phaseoloides* revealed 21 OSC sequences, among which eight unigenes were putative β-amyrin synthases. A full-length OSC sequence (EpBAS) with high identity to β-amyrin synthase was obtained (Additional file 8). The EpBAS cDNA included a 2289 bp full open reading frame fragment. The deduced amino acid sequence of EpBAS (762 amino acids) shared 89.37 and 89.34% similarity with β-amyrin synthase in *Abrus precatorius* (ApBAS) and GiBAS in *Glycyrrhiza inflata* (Fig. 6), respectively. The relatively high similarities of the EpBAS protein with other β-amyrin synthases suggest that this gene encodes β-amyrin synthase in *Entada phaseoloides*.

### CYP450s and UGTs

**Fig. 7:**
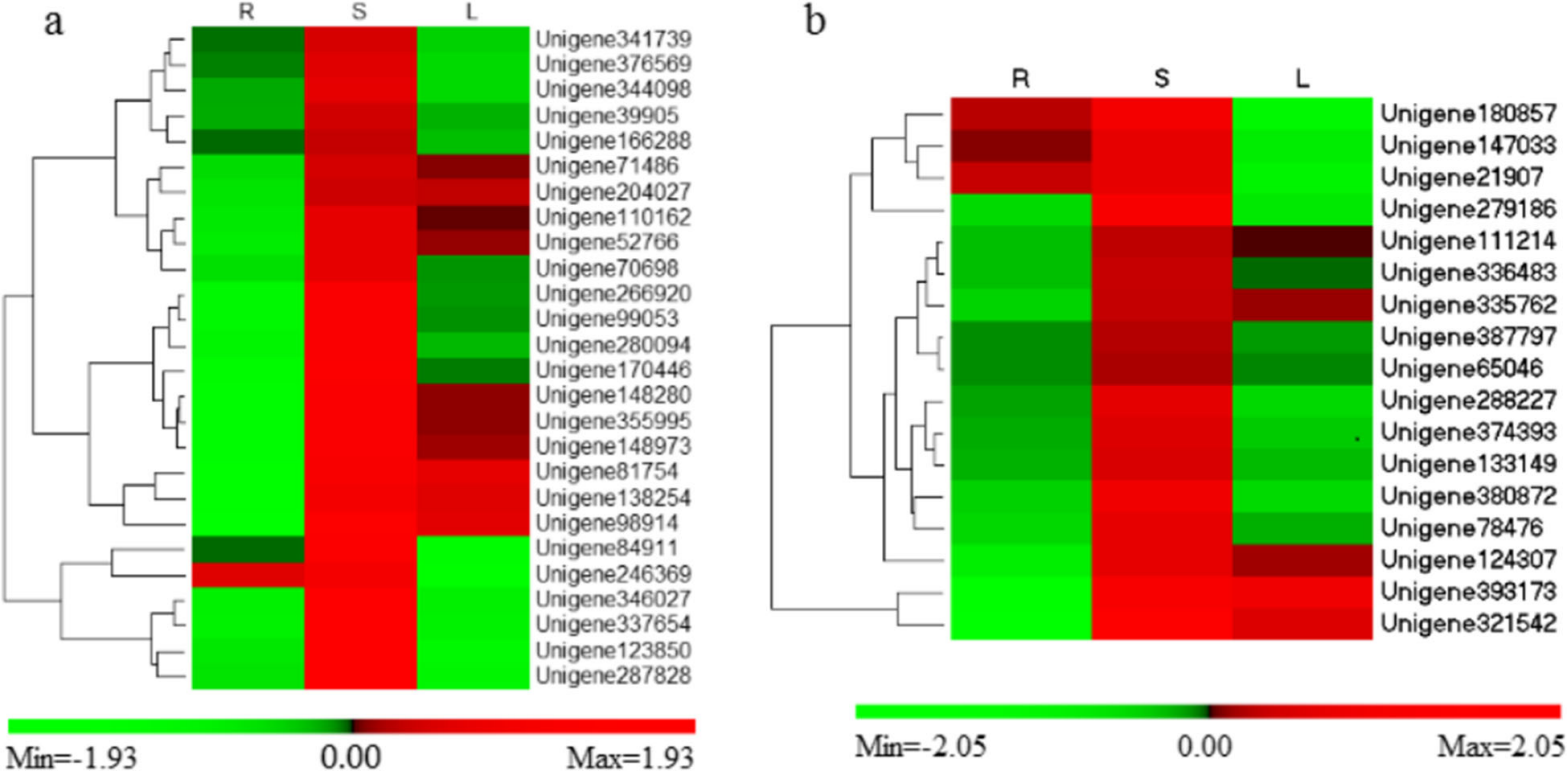
Heat map representing the upregulated unigenes of CYP450s (**a**) and UGTs (**b**) in stem (S) compared with the root (R) and leaf (L). The fold change expression data were obtained after three biological replicates

Earlier studies suggested that CYP450s and UGTs may account for the biosynthesis and accumulation of triterpene saponins in specific organs [34]. Tissue-specific transcriptome analysis of *Entada phaseoloides* suggests that the enzymes involved in triterpenoid saponin backbone are present in all the three tissues. Based on DEG analysis using transcriptome data, there is the possibility of further modifications such as oxidation and glycosylation using CYP450s and UGTs occur in the stem. Although the enzymes related to precursor biosynthesis are also present in root and leaf tissues which suggest the involvement of all the three tissues in the metabolic pathway. However, the metabolic analysis has indicated that it is the stem which mostly contains higher triterpenoid saponin content and utilized widely for its excellent pharmacological activity. In this study, in total of 326 CYP450s and 148 UGTs were found. Among the DEGs, 26 CYP450s and 17 UGTs were upregulated in the stem compared with the root and leaf tissues (Fig. 7a and b).

### qRT-PCR validation of candidate genes involved in triterpenoid saponin biosynthesis

**Fig. 8:**
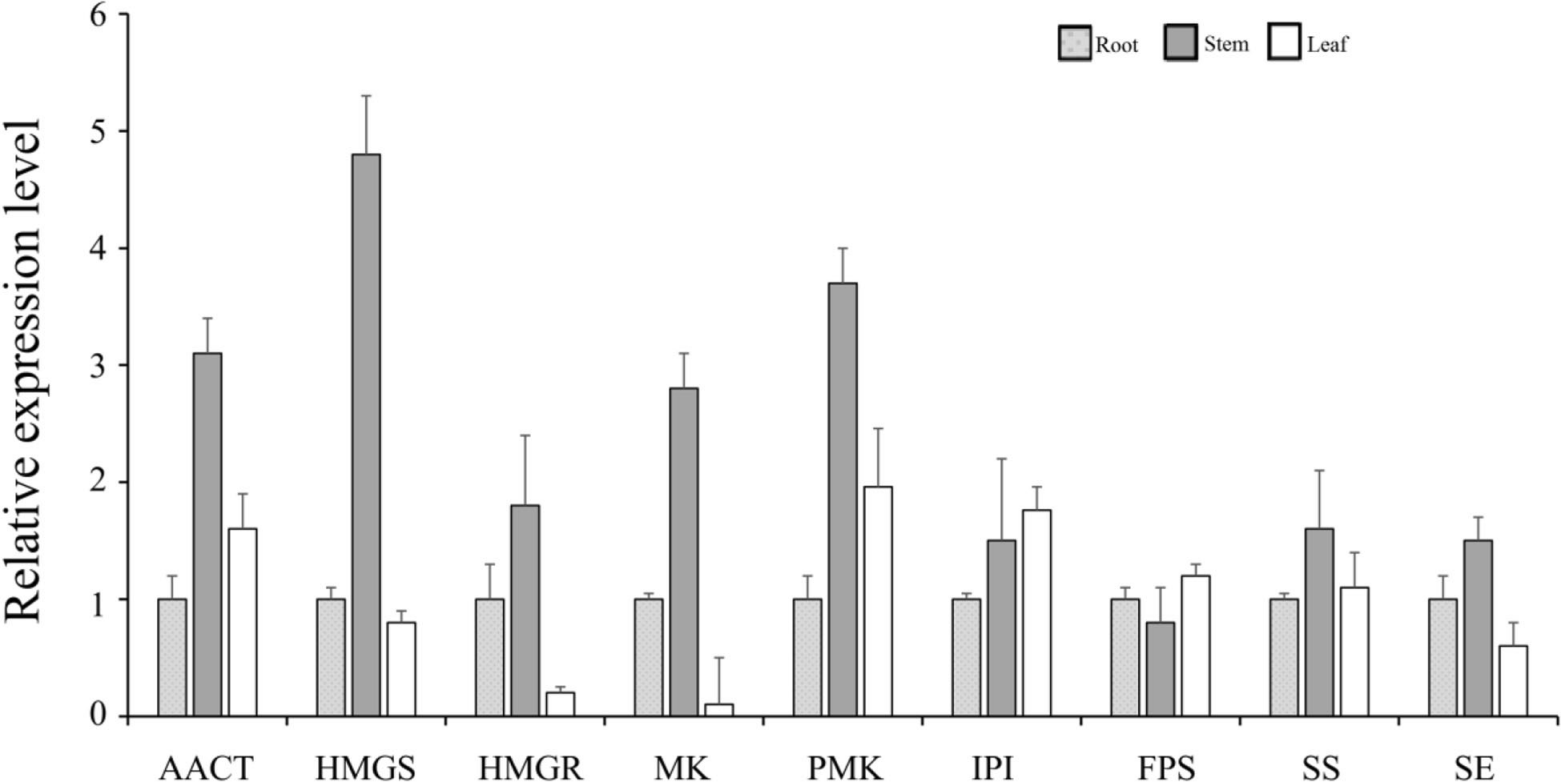
qRT-PCR validation of selected genes related to triterpene saponin biosynthesis. AACT: acetyl-CoA acetyltransferase; HMGS: hydroxymethyl- glutaryl-CoA synthase; HMGR: hydroxymethylglutaryl-CoA reductase; MK, mevalonate kinase; PMK: Phosphomevalonate kinase; IPI: isopentenyl pyrophosphate isomerase; FPS: farnesyl pyrophosphate synthase; SS: squalene synthase; SE: squalene epoxidase

To verify the expression profiles obtained from Illumina sequencing, we performed qRT-PCR on nine selected genes related to triterpene saponin biosynthesis (Fig. 8). Consistent with the Illumina data, most of these genes showed strong expression levels in the stem compared with the root and leaf, and acetyl-CoA acetyltransferase, hydroxymethylglutaryl-CoA synthase, hydroxymethylglutaryl-CoA reductase and SQE genes were expressed abundantly. The expression fold changes were also close to the RNA-seq results. qRT-PCR results indicate that the RNA-seq data in this studty were reliable.

## Discussion

*Entada phaseoloides* is an important traditional medicinal plant with various pharmaceutical activities. Although this plant is pharmacologically important, its genomic or transcriptomic information is highly limited. In NCBI, only 38 protein sequences are accessible for *Entada phaseoloides*. We revealed the comparative transcriptome analysis of the root, stem, and leaf tissues of *Entada phaseoloides*. The dataset reported here is useful in understanding the biosynthetic pathway of pharmacodynamic triterpenoid saponin and genetic engineering of this species.

In this study, a total of 53.26 Gb clean data were generated from nine RNA-seq libraries of the root, stem, and leaf. De novo assembly acquired 116,910 unigenes, with an average N50 length of 1218 bp, which is similar to that of previously reported non-model plants, such as *Raphanus sativus* [35] and *Isodon Amethystoides* [36]. The best match for each unigene search against the Nr and KEGG databases was of help to assign GO functional annotation under biological process, cellular component, and molecular function categories. The varied GO assignments to unigenes represented the possible assortment of genes in the *Entada phaseoloides* transcriptome. Several unigenes mapped onto KEGG are related to distinct secondary metabolic pathways. Most unmatched unigenes are short sequence proteins with no domain, untranslated regions, non-coding RNA or assembly mistakes. In support of the annotation, all the unigenes encoding enzymes related to the upstream regions of the MVA pathway for saponin biosynthesis from acetyl-CoA to squalene were found.

SQE enzymes catalyze the oxidation of squalene to 2,3-oxidosqualene. In our transcriptomic analysis, sequences encoding SQE represented the highest number (16) of unigenes associated with the MVA pathway. Single copies of SQE were identified in mouse and yeast, and the destruction of SQE in these species is lethal [37]. However, two or more copies of SQE are usually found in plants. Hwang et al. [38] examined 17 SQE sequences in *Eleutherococcus senticosus.* In *Arabidopsis thaliana*, six SQE enzymes have been identified, and three of them encode functional SQEs [39]. The expression of PgSQE1 regulates the biosynthesis of ginsenoside in *Panax ginseng* [40]*.* Thus, SQE is possibly an important enzyme in the saponin biosynthetic pathway. The SQE enzyme responsible for the saponin biosynthesis in the 16 SQE sequences in *Entada phaseoloides* remains to be identified.

The cyclization of 2,3-oxidosqualene is a branch point of saponin synthesis. The major saponins in the stem of *Entada phaseoloides* are oleanane-type triterpenoids. Oleanolic acid sapogenin was derived from β-amyrin after hydroxylation by CYP450s and glycosylation by UGTs [41, 42]. A total of 8 β-amyrin synthase, 326 CYP450, and 148 UGT sequences were observed in our transcriptome. The high expression of β-amyrin synthase, an important enzyme related to triterpenoid sapogenin biosynthesis at later stages, further reveals the high concentration of sapogenins in the stem of *Entada phaseoloides*. Similar tissue-specific concentrations of triterpenoid sapogenins have already been reported in other plants [43–45]. Moreover, 26 CYP450s and 17 UGTs were found to be upregulated in the stem. Further characterization of these candidate enzymes is needed to confirm the pathway of triterpenoid saponin biosynthesis in *Entada phaseoloides.*

## Conclusions

In the present study, the comparative transcriptome analysis of root, stem and leaf tissues of *Entada phaseoloides* was performed to investigate the putative genes involved in triterpenoid saponin biosynthetic pathway of an important medicinal plant. The differential expression pattern of pathway genes suggest tissue-specific synthesis. The identified data will help the further discovery and functional genomics and transcriptomics analysis of *Entada phaseoloides*.

## Methods

### Plant materials

Three-year-old healthy wild-type *Entada phaseoloides* plants were collected from the experimental farm of South China Botanical Garden, Guangzhou City, Guangdong Province, P.R. China, in May 2019. After cleaning with ultrapure water, the roots, stems, and leaves were collected separately, immediately frozen in liquid nitrogen, and stored at − 80 °C.

### RNA extraction, cDNA synthesis, and sequencing

Total RNA from approximately 1.0 g of each tissue was extracted using TRIzol (Invitrogen, Canada) following the manufacturer’s instructions. Three replicates were employed for each experiment. mRNA was isolated from total RNA by using Oligo (dT) magnetic beads. By mixing with fragmentation buffer, the mRNA was broken into short fragments.

The short fragments were purified and resolved with EB buffer. After end repair and single base “A”addition, adapters were ligated to the cDNA molecules. To select suitable cDNA fragments for PCR amplification, we purified the sample library with the AMPure XP system (Beckman Coulter, USA). Finally, PCR products were purified, and library quality was were determined using an Agilent Bioanalyzer 2100 system (Agilent Technologies, USA) and a Qubit 3.0 fluorometer (Invitrogen, USA). Each cDNA library was sequenced in a single lane of the Illumina Hiseq 2500 platform.

### Data filtering and de novo assembly

The raw reads were first filtered to exclude the reads containing adaptors or with ambiguous nucleotides (‘N’). Next, the low-quality reads having more than 20% Q < 20 bases were also trimmed. The yielded high-quality clean reads were used to the develop sequence assembly by using the Trinity software. After the removal of redundant Trinity-generated sequences by using the TGICL, clusters and unigenes were finally obtained.

### Functional annotation and classification

All assembled unigenes were annotated by BLASTx analysis against the Nr (http://www.ncbi.nlm.nih.gov/), UniProt (http://www.uniprot.org/downloads), Pfam (http://pfam.xfam.org/), COG (http://www.ncbi.nlm.nih.gov/COG/) databases with an E-value <1e-5. Only the top hit results were extracted for each unigene. GO (http://www.geneontology.org) terms were functionally classified based on Nr annotations by using the Blast2go program (http://www.Blast2 go.de/). KEGG (http://www.genome.jp/kegg/) was used to draw metabolic maps. The KEGG analysis results included KEGG orthology (KO) numbers and enzyme commission (EC) numbers.

### DEG analysis

Clean reads were mapped back onto the assembled unigenes by using the BWA program. The FPKM value was calculated for each unigene in each tissue of *Entada phaseoloides*. The expression difference was analyzed by Fisher’s exact test, and the FDR for each gene were obtained. DEGs were required to have thresholds of FDR ≤ 0.001 and |log2Ratio| ≥ 1. KEGG pathways were then reconstructed on DEGs.

### Verification of gene expression by using qRT-PCR

Nine genes related to triterpene saponin biosynthesis were selected for validation by qRT-PCR. The primers for qRT-PCR analysis are listed in Additional file 9. All reactions were performed on the CFX96 real-time PCR system (Bio-Rad, USA) with a SYBR® Premix Ex Taq™ kit (Takara, China). The *Actin* gene was used as an internal control (Additional file 10). Each qRT-PCR experiment was performed with three biological repeats. The relative gene expression was calculated using the 2^−ΔΔCT^ method.

## Supplementary information

**Additional file 1.** Summary of transcriptome sequencing and assembly results.

**Additional file 2.** Correlation indices between different samples.

**Additional file 3. **Functional annotation of *Entada phaseoloides* unigenes.

**Additional file 4.** Frequencies of unigenes matching GO terms.

**Additional file 5.** GO enrichment of unigenes.

**Additional file 6.** Unigenes for KEGG analysis.

**Additional file 7.** DEGs in different tissues.

**Additional file 8. **Sequence of β-amyrin synthase (EpBAS) gene in *Entada phaseoloides*.

**Additional file 9.** List of qRT-PCR primer sequences.

**Additional file 10. **Melting curves of reference gene *Actin* for qRT-PCR amplification.

## Data Availability

Final sequences obtained from all the three tissues were submitted to the SRA database of NCBI with accession number PRJNA597694.

## Abbreviations

MVA: mevalonic acid
IPP: isopentenyl diphosphate
SQE: squalene epoxidase
OSC: oxidosqualene cyclase
CYP450: Cytochrome P450 monooxygenase
UGT: UDP-glycosyltransferase
TGICL: The TGI clustering tool
Nr: Non-redundant
KEGG: Kyoto Encyclopedia of Genes and Genomes
GO: Gene Ontology
COG: Clusters of Orthologous Groups
DEG: Differentially expressed gene
BAS: β-amyrin synthases
qRT-PCR: Quantitative reverse transcription-polymerase chain reaction
