# Supplementary material for: Comparative transcriptome analysis of root, stem, and leaf tissues of Entada phaseoloides reveals potential genes involved in triterpenoid saponin biosynthesis
Source: BMC Genomics. 2020 Sep 15;21:639. doi: 10.1186/s12864-020-07056-1 (PMC7493163; doi:10.1186/s12864-020-07056-1)
Supplement: Supplementary file 1 — Additional file 1. Summary of transcriptome sequencing and assembly results. [file 12864_2020_7056_MOESM1_ESM.doc]

**Supplementary file 1.** Summary of transcriptome sequencing and assembly results.

|  | Root | Stem | Leaf |
| --- | --- | --- | --- |
| Clean data | 8673312900 | 8541007500 | 8865692100 |
| Q30 percentage | 93.88% | 94.16% | 94.05% |
| Clean reads | 57822086 | 56940050 | 59104614 |
| Total transcripts | 156110 | | |
| Mean length | 1051 | | |
| N50 | 1218 | | |
| Percentage GC | 46.26% | | |
