# Supplementary figures and images for: Comparative transcriptome analysis of root, stem, and leaf tissues of Entada phaseoloides reveals potential genes involved in triterpenoid saponin biosynthesis
Source: BMC Genomics. 2020 Sep 15;21:639. doi: 10.1186/s12864-020-07056-1 (PMC7493163; doi:10.1186/s12864-020-07056-1)

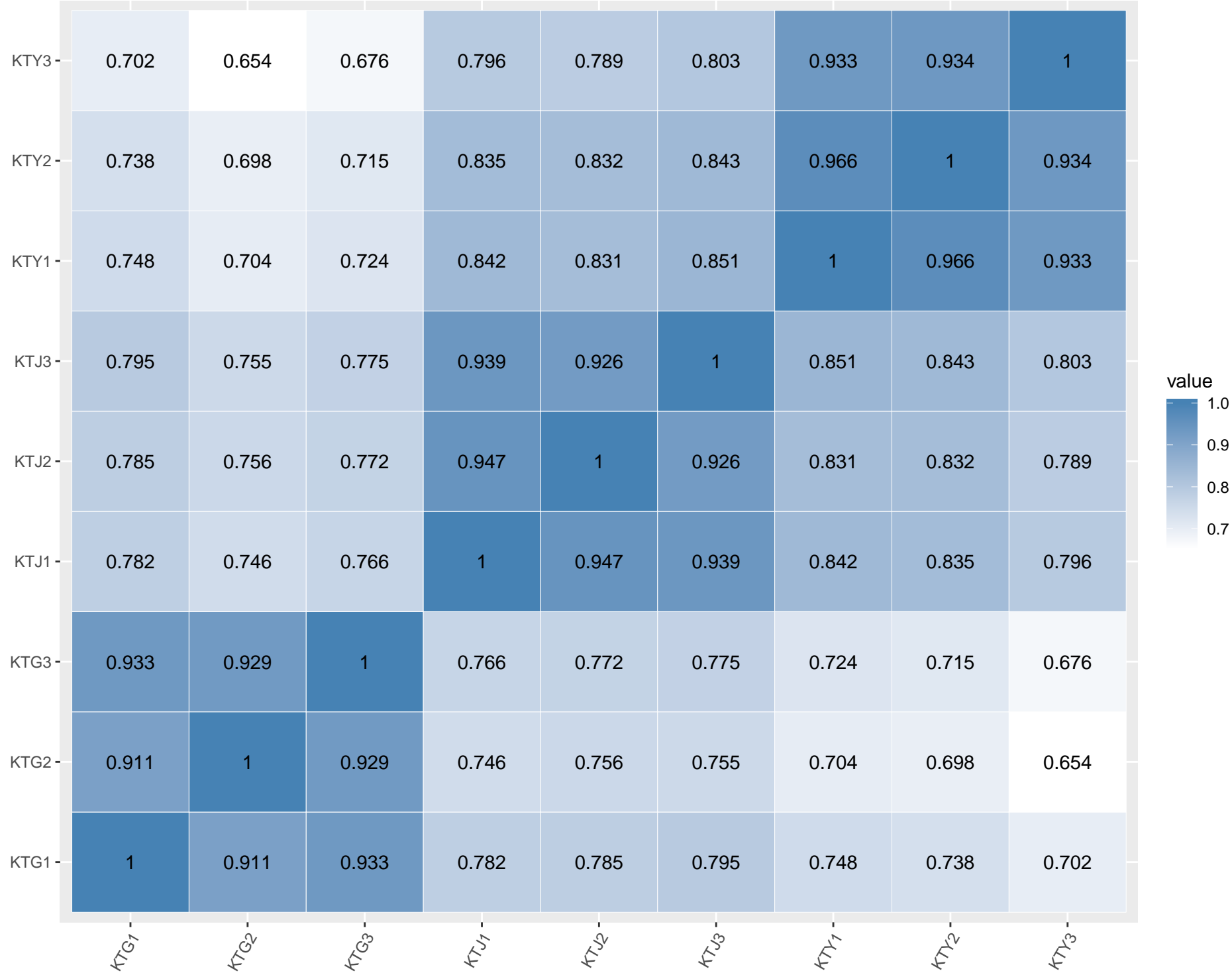

Supplement: Supplementary file 2 — Additional file 2. Correlation indices between different samples. [file 12864_2020_7056_MOESM2_ESM.pdf]

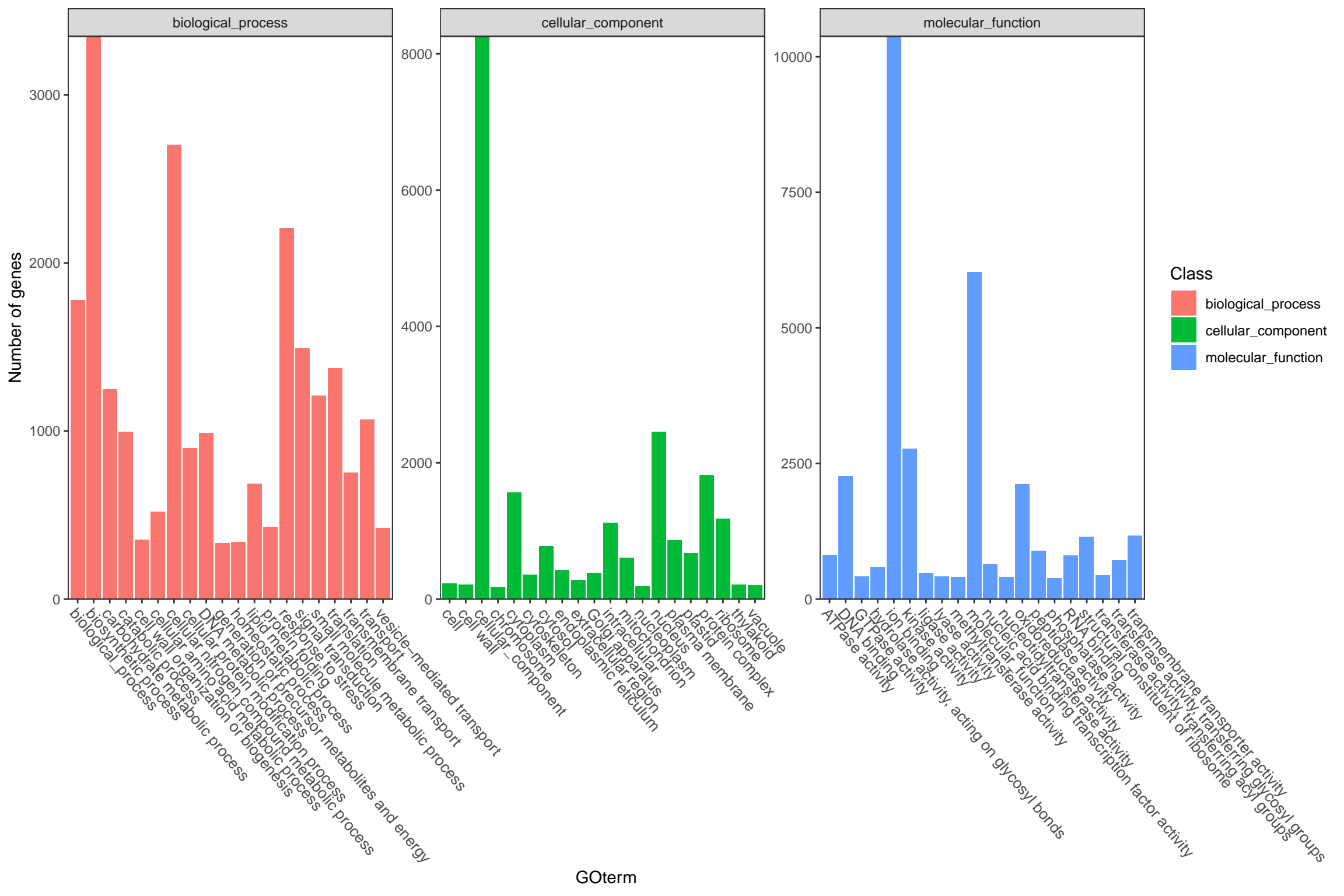

Supplement: Supplementary file 4 — Additional file 4. Frequencies of unigenes matching GO terms. [file 12864_2020_7056_MOESM4_ESM.pdf]

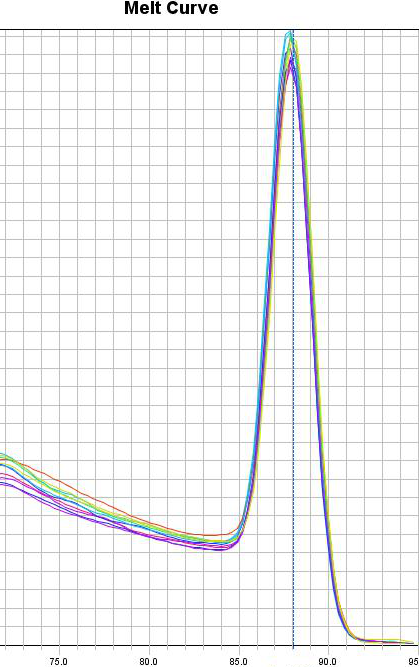

Supplement: Supplementary file 10 — Additional file 10. Melting curves of reference gene Actin for qRT-PCR amplification. [file 12864_2020_7056_MOESM10_ESM.tif]
