# Supplementary material for: Comparative transcriptome analysis of root, stem, and leaf tissues of Entada phaseoloides reveals potential genes involved in triterpenoid saponin biosynthesis
Source: BMC Genomics. 2020 Sep 15;21:639. doi: 10.1186/s12864-020-07056-1 (PMC7493163; doi:10.1186/s12864-020-07056-1)
Supplement: Supplementary file 9 — Additional file 9. List of qRT-PCR primer sequences. [file 12864_2020_7056_MOESM9_ESM.docx]

**Supplementary file 9.** List of qRT-PCR primer sequences.

| **Target genes** |  | **Primer sequences (5′ to 3′)** |
| --- | --- | --- |
| Acetyl-CoA acetyltransferase | F | TGCAGCAGCACCATCACTTA |
|  | R | TACGCAAACTCCGACCAAGT |
| Hydroxymethylglutaryl-CoA synthase | F | TGCTGAACACAGGTGGAAGG |
|  | R | CAAGGGAGCGCCTATTGGAT |
| Hydroxymethylglutaryl-CoA reductase | F | GTTCGGACAAGAAAGCAGCG |
|  | R | TTCCACGCTGGTCTTCAACA |
| Mevalonate kinase | F | AACACCAGCCACCAATGCT |
|  | R | CAGCCTCAAGTCTGGTGAGTTT |
| Phosphomevalonate kinase | F | ACCAGTTCCCGGTTCTCCTA |
|  | R | TGCAGTGCCTGTACCAGAAG |
| Isopentenyl pyrophosphate isomerase | F | GCTGCATAGAGCTTTCAGCG |
|  | R | ACCTTTGTTGCAGATCGTTGC |
| Farnesyl pyrophosphate synthase | F | AAGCAGTCTGGAACTCGACC |
|  | R | TGAGGTTGTACAAATGTCAGGGT |
| Squalene synthase | F | GATGGGTGCAGGAATGGCTAA |
|  | R | TAAACCCAGCCCAACAAGTCC |
| Squalene epoxidase | F | TTCTACACCCTACGCAAGCC |
|  | R | GCTGGATCAGGAGATGCACA |
| Actin | F | TTGGACTGTGCCTCATCACC |
|  | R | CTTCCATCACCCTCGGCATT |
